# Array MEMS Vector Hydrophone Oriented at Different Direction Angles

**DOI:** 10.3390/s19194282

**Published:** 2019-10-03

**Authors:** Mengran Liu, Lei Nie, Shanqiang Li, Wen Jia, Lansheng Zhang, Guojun Zhang, Wendong Zhang

**Affiliations:** 1 Hubei Key Laboratory of Modern Manufacturing Quantity Engineering, School of Mechanical Engineering, Hubei University of Technology, Wuhan, Hubei 430068, China; liumengran1991@163.com (M.L.); 15207174234@163.com (S.L.); jiawen_1995@163.com (W.J.); 2 Science and Technology on Electronic Test and Measurement Laboratory, North University of China, Taiyuan 030051, China; 18734877016@163.com (L.Z.); zhangguojun1977@nuc.edu.cn (G.Z.); wdzhang@nuc.edu.cn (W.Z.)

**Keywords:** MEMS, array vector hydrophone, left-right ambiguity, different direction angles, Position

## Abstract

A new type of array MEMS (Microelectro Mechanical Systems) vector hydrophone has been proposed to solve the left-right ambiguity problem that is commonly found in current ones. Meanwhile, the advantages of good sensitivity and low fabrication cost are maintained. The array MEMS vector hydrophone is integrated by four units oriented at different direction angles. By the aid of this kind of vector hydrophone, not only the exact direction of the sound source can be measured, but also the position obtained. The working principle of the array microstructure has been analyzed and simulated. The result shows that the position of the sound source can be well determined. The prototype of the hydrophone is fabricated based on standard MEMS technology, and its performance is tested in a standing wave tube and an anechoic tank. The testing results show that the array hydrophone exhibits a good consistency of all the four units and satisfactory performance. More importantly, this array hydrophone exhibits excellent ability of positioning with the relatively small angle error. Thus, a MEMS hydrophone with multiple functions and relatively high performance is realized, which has important theoretical and practical significance in relevant applications such as the small-size underwater vehicles.

## 1. Introduction

Sound wave is the main information carrier for underwater long-distance communication. Therefore, the ocean research and exploration mainly rely on underwater acoustic technology and one of the key tools is the sonar [1]. For the sonar, the hydrophone is the core part and its performance obviously affects the positioning ability.

Hydrophones can be categorized into two kinds: scalar and vector. The scalar hydrophone can only measure the sound pressure, which means that the direction of the sound source cannot be estimated by a single scalar hydrophone. By contrast, the vector one can simultaneously measure the sound pressure and gradient information, so that the direction can be estimated [2]. There are two types of traditional vector hydrophones: co-vibrating and sound pressure gradient. However, the co-vibrating hydrophones are easily affected by the suspension system, and sound pressure gradient hydrophones have poor sensitivities at low frequency [3,4]. Moreover, due to their relatively large volume, these two types of hydrophones are both difficult to be integrated into the small-size underwater vehicle.

With the development of MEMS technology, the new MEMS hydrophones with the advantages of miniaturization, integration and mass production have been proposed, which are very suitable for the small-size underwater vehicle [5,6]. A kind of MEMS vector hydrophone with cilium-four-beam microstructure was proposed by Zhang [7], which has a good sensitivity at low-frequency. Although great progresses have been made after years of optimization [8,9,10], the problem of left-right ambiguity is kept unsolved. That is to say, the exact direction cannot be found. An improved composite MEMS hydrophone in which a capacitor microstructure has been integrated near the cilium-four-beam was proposed in previous work [11]. However, the sensitivity of sound pressure part (capacitor) is lower than the vector part (cilium-four-beam). Therefore, the detection performance of the hydrophone is impaired. Moreover, this MEMS composite hydrophone consisting of two parts leads to complex process and high cost.

Therefore, to get rid of the disadvantages caused by the capacitor, this paper proposes an array MEMS hydrophone with four cilium-four-beam microstructures oriented at different direction angles. Because these microstructures have similar structures, the fabrication is relatively easy and economical. Because each microstructure has an independent coordinate, this array hydrophone can simultaneously measure the different direction information. By this way, not only the left-right ambiguity is avoided, but also the exact position of the sound source can be obtained.

## 2. Working Principle of MEMS Vector Hydrophone

**Figure 1:**
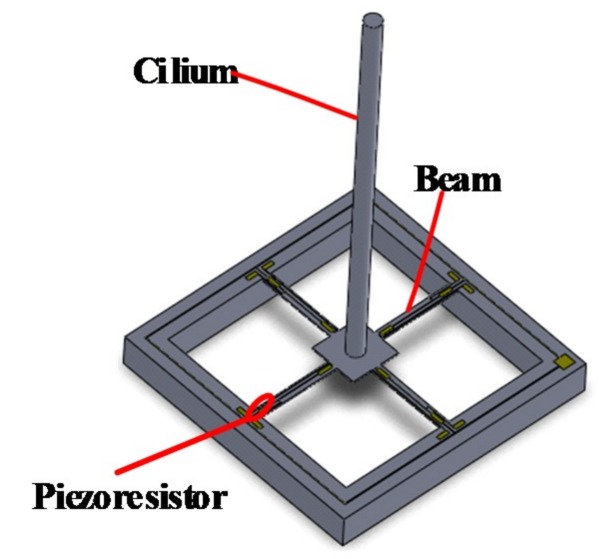
The sensing microstructure of the MEMS vector hydrophone.

**Figure 2:**
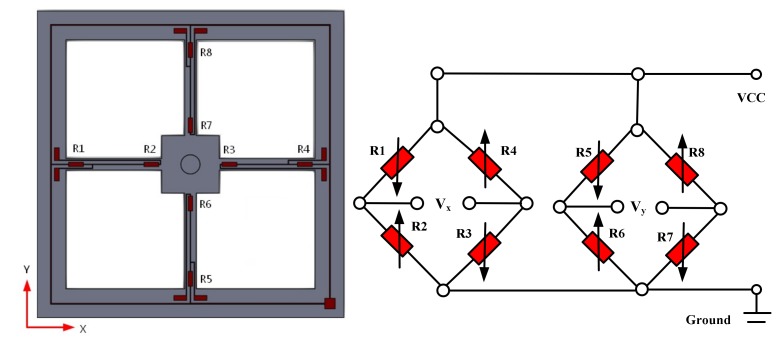
Wheatstone bridges.

The sensing microstructure of the MEMS vector hydrophone is shown in Figure 1. It consists of the four-beam and the cilium which is vertically fixed at the center of the four-beam. Eight equivalent piezoresistors R1–R8 are distributed on the four-beam. R1–R4 and R5–R8 respectively constitute two Wheatstone bridges, as shown in Figure 2. The sound signals act on the cilium and make it deflected, which causes the four-beam deformed and the values of the piezoresistors changed. And then the voltages in the X and Y directions (Vx and Vy) are obtained by Wheatstone bridges.

**Figure 3:**
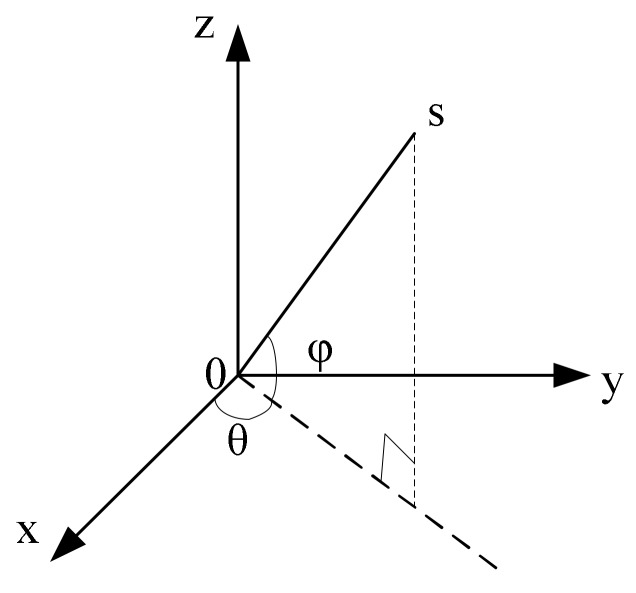
The directional measurement model of MEMS vector hydrophone.

The directional measurement model of MEMS vector hydrophone is shown in Figure 3, in which S denotes the sound source, θ is the horizontal angle in the range of [0, 2π], and φ is the pitch angle in the range of [0, π]. Any point in the sound vector field can be decomposed into particle vibration velocity *v*(*r*,*t*) and sound pressure *p*(*r*,*t*), where t represents time, and r is the distance between the point and the sound source.

According to the fundamental law of sound wave, Euler equation [12,13], the relation between sound pressure *p*(*r*,*t*) and vibration velocity *v*(*r*,*t*) at any point can be expressed as:

(1) $$\frac{\partial v(r,t)}{\partial t}+\frac{1}{\rho }\nabla p(r,t)=0,\;\mathrm{that}\;\mathrm{is},\;v(r,t)=-\frac{1}{\rho }\int \nabla p(r,t)dt$$

where ∇ and *ρ* denote gradient operator and medium density, respectively.

The sound pressure of plane wave can be seen as the superposition of harmonic plane waves, and thus Equation (1) can be expressed as:

(2) $$v(r,t)=-\frac{1}{\rho }\iint \nabla \left[X(\omega )e^{j(\omega t-kr)}\right]d\omega dt$$

where *k* and *ω* are wave constant number and angular frequency respectively.

Integrating over time t, the following equation can be obtained.

(3) $$\begin{array}{c} v(r,t)=-\frac{1}{\rho }\int \nabla \left[\frac{X(\omega )}{j\omega }e^{j(\omega t-kr)}\right]d\omega \\ =\frac{1}{\rho c}\left[\cos\theta \cos\varphi \cdot \xi +\sin\theta \cos\varphi \cdot \eta +\sin\varphi \cdot \zeta \right]p(r,t) \end{array}$$

where *ξ, η, ζ* are the unit vector in X,Y and Z direction respectively, and *ρc* denotes wave impedance (c is velocity of the sound wave in the media) which is a real number in the plane wave sound field. The vibration velocity of the medium particle *v*(*r*,*t*) in the X,Y and Z direction are respectively *v_x_*(*r*,*t*), *v_y_*(*r*,*t*) and *v_z_*(*r*,*t*), and then:

(4) $$\left\{ \begin{array}{l} p(r,t)=\rho cv(r,t)\\ v_{x}(r,t)=v(r,t)\cos\theta \cos\varphi \\ v_{y}(r,t)=v(r,t)\sin\theta \cos\varphi \\ v_{z}(r,t)=v(r,t)\sin\varphi \end{array}\right.$$

In the far-field approximation, *φ* = 0. The outputs of the MEMS vector hydrophone *V_x_* and *V_y_* are proportional to the vibration velocity component *v_x_*(*r*,*t*) and *v_y_*(*r*,*t*). Thus, as long as the outputs of the hydrophone are measured, the horizontal angle *θ* can be obtained as the following.

(5) $$\theta =\arctan(\frac{V_{y}}{V_{x}})$$

These are the basic directional principles of a single MEMS vector hydrophone. However, the outputs of the MEMS vector hydrophone *V_x_* and *V_y_* are positive values. Therefore, the single sensing microstructure cannot determine the precise direction (positive or negative angle) of the sound source. That is to say, it has the problem of left-right ambiguity. Moreover, when the direction of sound source is nπ/2 (n = 0, 1, 2 and 3), *V_x_* or *V_y_* should tend to be zero. Such results are difficult to be obtained due to the noises.

## 3. Design of Array MEMS Hydrophone

To solve the above problems, the array MEMS hydrophone with four different direction-angle sensing units integrated onto the same chip has been proposed. The working principle of such structure is explained as follows. When the microstructure of the array MEMS vector hydrophone perceives the sound signal from sound source S, each sensing unit will have two outputs -Vx and Vy, and generate two angle lines θn and θn′ in its own relative coordinate system XnOnYn (n = 1, 2, 3 and 4). Then, the slope-intercept form y = kx + b of every angle line in the absolute coordinate system XOY can be calculated. If four such lines, in which every line comes from angle line θn or θn′ (n = 1, 2, 3 and 4) intersect at the same point, then the intersection point is considered as the position of sound source.

**Figure 4:**
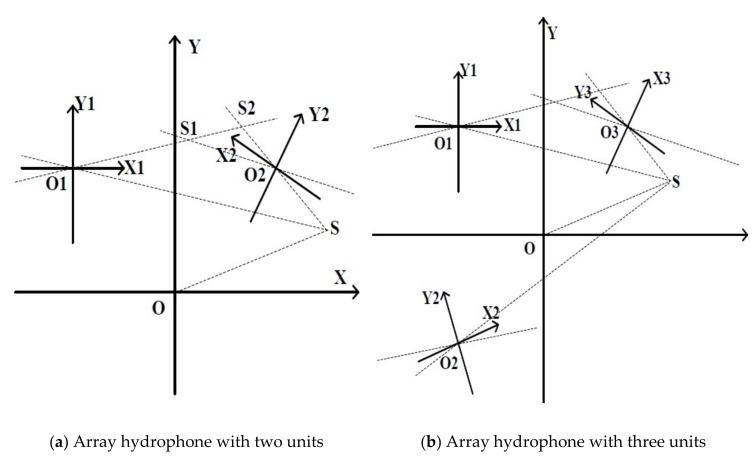
The positioning results of the array MEMS vector hydrophone.

In fact, from the perspective of positioning function, the number of units integrated onto the same chip should be at least 3. For brevity, the positioning results of the array MEMS vector hydrophone integrated with two units (Figure 4a) and three units (Figure 4b) have been illustrated, respectively. From Figure 4, it can be seen that the two-unit one will misjudge the position of the sound source S for generating intersections S1 and S2, which are false targets. And the sound source can be positioned by the array hydrophone with at least three units because of the unique intersection point coming from three angle lines in three units respectively.

**Figure 5:**
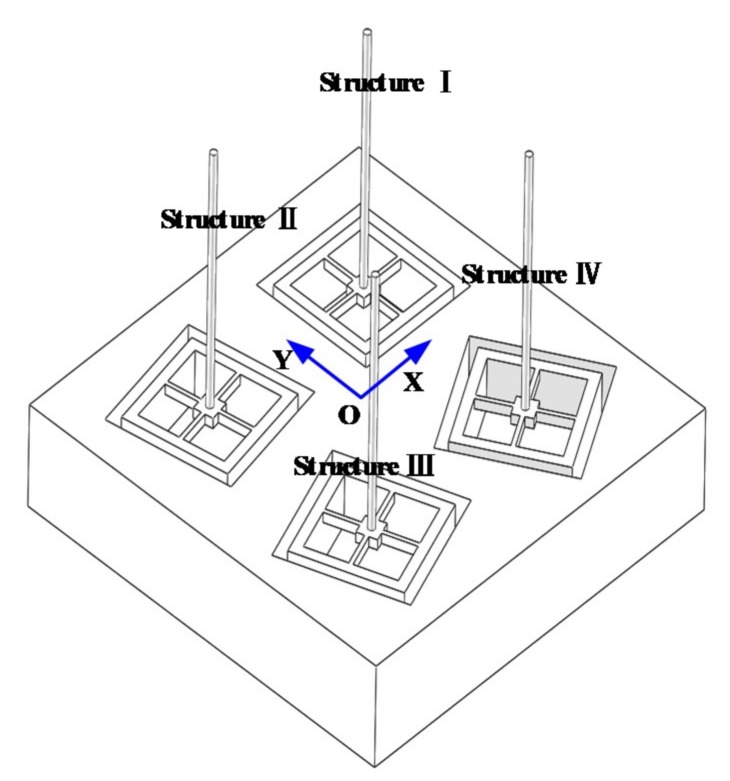
Overall design of the array hydrophone.

**Figure 6:**
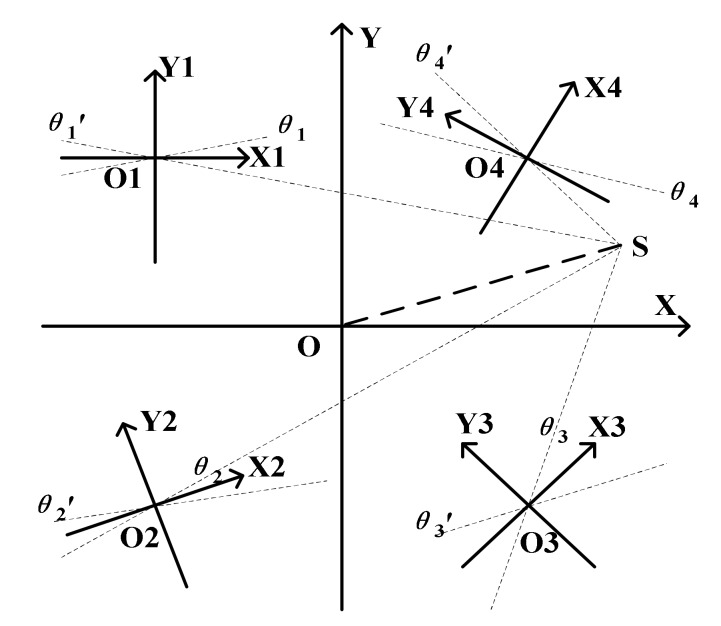
The positioning principle of array hydrophone.

Considering that the volume and cost of four-unit array hydrophone are similar to that of three-unit array hydrophone and the positioning accuracy and reliability of four-unit sensor are higher in practical application, the array MEMS vector hydrophone with four different direction-angle units is designed. It consists of structure I, structure II, structure III and structure IV, which are respectively rotated 0°, 30°, 45° and 60° counterclockwise to form the new relative coordinate systems XnOnYn (n = 1, 2, 3 and 4), as shown in Figure 5. The positions of the four units in absolute coordinate are respectively (−8, 8), (−8, −8), (8, −8) and (8, 8), whose units are mm. The positioning principle of array hydrophone is shown in Figure 6.

**Figure 7:**
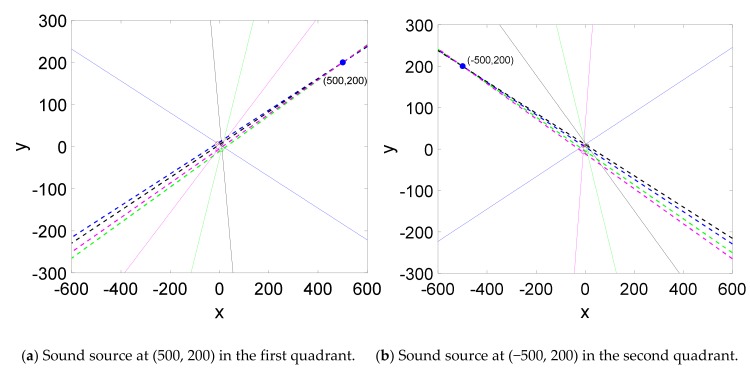

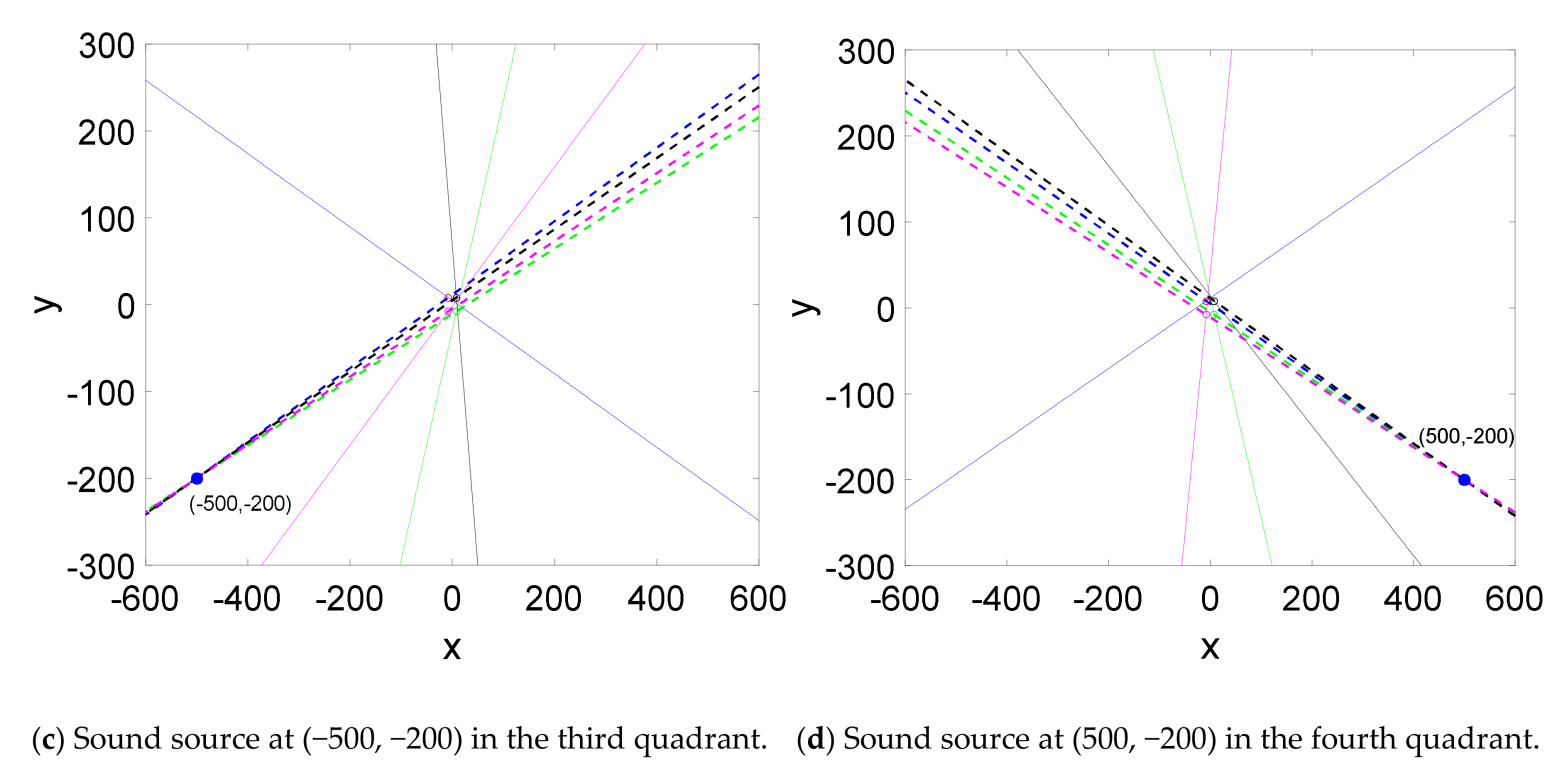
Simulated results of the array hydrophone.

The positioning model of the array hydrophone was established in MATLAB, and the positioning simulations were carried out to measure the position of the sound sources (500, 200), (−500, 200), (−500, −200) and (500, −200) which are located in the four quadrants respectively. The positioning results are shown in Figure 7a–d. It can be concluded that the array hydrophone can realize the accurate position of sound source.

## 4. Fabrication 

**Figure 8:**
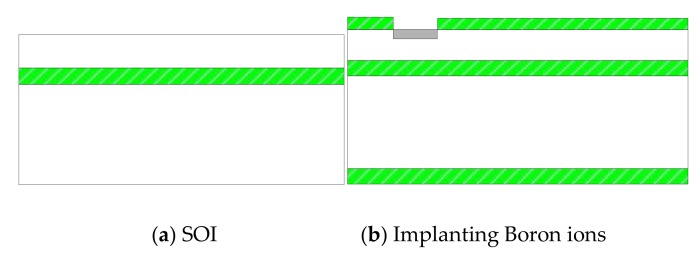

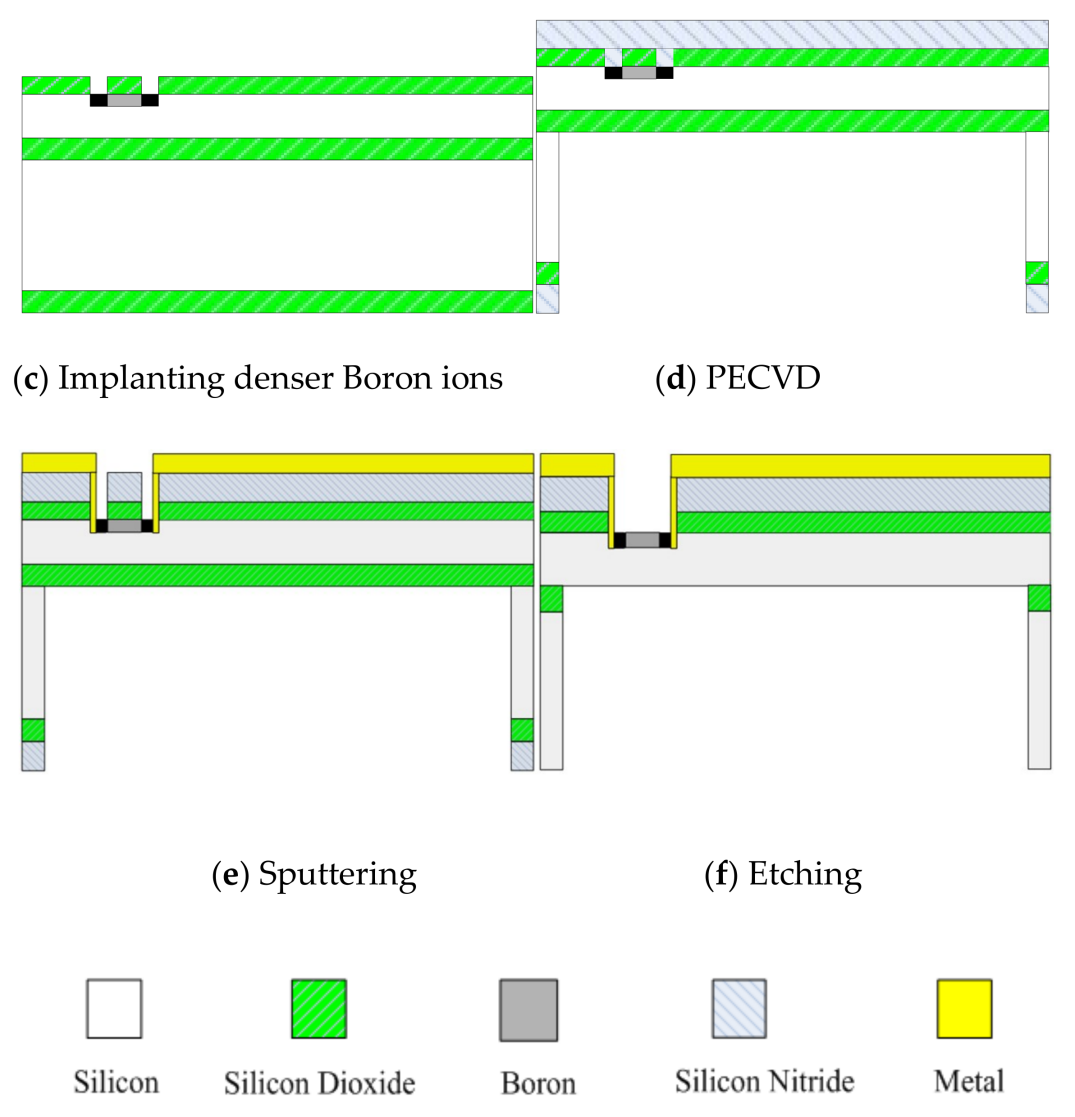
The processing technology of the four-beam microstructure.

**Figure 9:**
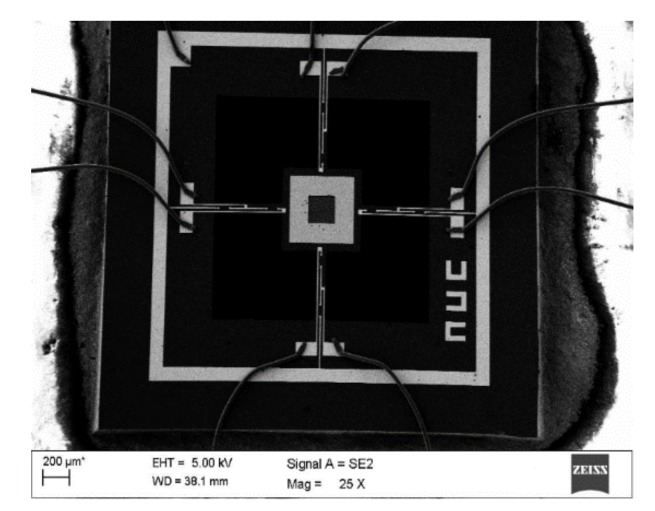
SEM diagram of MEMS four-beam microstructure.

**Figure 10:**
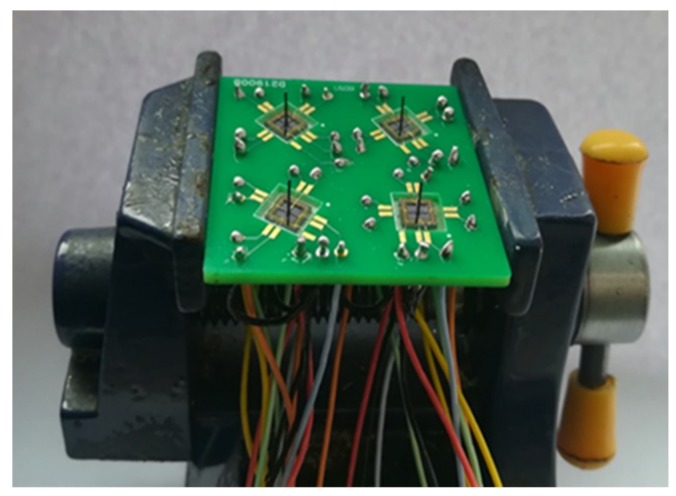
The fabricated array MEMS vector microstructure.

It is difficult to directly fabricate the cilium-four-beam sensing unit because of the high aspect ratio, so four-beam microstructure has to be first completed. Its processing technology is shown in Figure 8a–f [14,15]: (a) Preparing the N-type SOI (Silicon-on-Insulator) chip with 40 μm device layer; (b) Forming the 1000 Å silicon dioxide film at 950 °C, etching by RIE (reactive ion etching) and implanting Boron ions to form piezoresistors; (c) Forming the silicon dioxide film again, etching by RIE and implanting the denser Boron ions, and then annealing to activate the piezoresistors and form ohmic contacts; (d) Depositing double-sided silicon nitride by PECVD (plasma enhanced chemical vapor deposition), etching the backside silicon nitride and silicon dioxide by RIE, and etching the substrate silicon by DRIE (deep reactive ion etching); (e) Sputtering 200Å Cr to form an adhesion layer and 1000Å Au, and etching Au and Cr to form the Wheatstone bridge; (f) etching silicon nitride and silicon dioxide by RIE, and etching device layer silicon by DRIE to release four-beam microstructure. The SEM (scanning electron microscope) diagram of four-beam microstructure is shown in Figure 9. Considering the design requirements and processing costs, PCB board is used as the substrate to package the chip. The cilia (plastic fiber cylinders) with same lengths (4mm) are integrated to the centers of the four-beam micro-structure by automatically integrated system [16]. The fabricated array MEMS vector microstructure is shown in Figure 10.

## 5. Performance Tests

To protect the MEMS microstructure and, simultaneously, to ensure good acoustic signal transmission, the MEMS microstructure is packaged inside a nitrile butadiene rubber (NBR) sound-transparent cap, filled with silicon oil [17]. The performance tests of the array MEMS vector hydrophone include sensitivity test, directivity test and positioning test. In the tests, the array hydrophone and the reference hydrophone (RS-100) with sensitivity of −180 dB (0 dB ref 1 V/Pa) and bandwidth of 20–100 kHz are generally placed on the same horizontal surface.

### 5.1. Sensitivity Test

**Figure 11:**
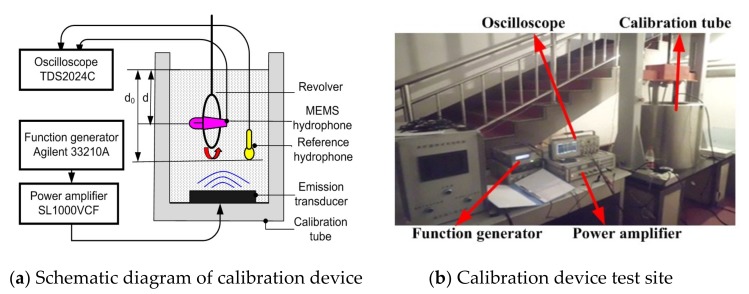
The diagram of calibration device and its illustration.

Sensitivity test and directivity test are completed in standing wave calibration tube as shown in Figure 11. The tested array MEMS hydrophone is elastically suspended on the revolver, and the reference hydrophone is placed in the calibration tube.

**Figure 12:**
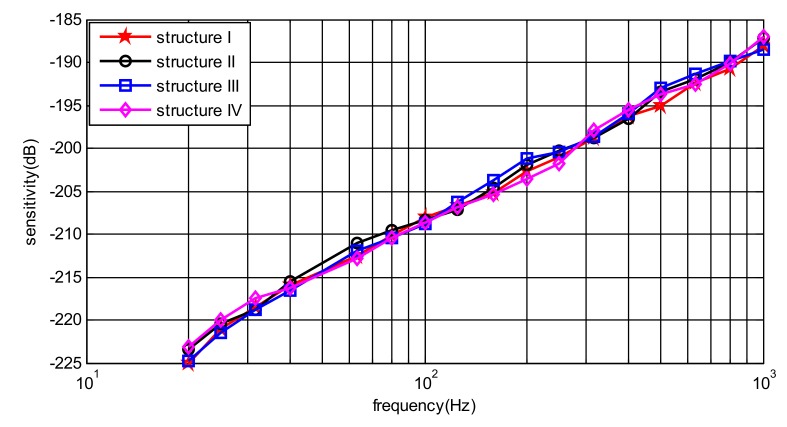
Sensitivity curves of the array MEMS hydrophone in *Xn* direction.

The sensitivity test of the array MEMS hydrophone is completed by comparison calibration method [18]. The outputs of the MEMS hydrophone and the reference hydrophone should be simultaneously recorded. The sensitivity of the tested MEMS hydrophone *M_n_* (n = 1, 2, 3 and 4) is shown in Equation (6).

(6) $$M_{n}=M_{0}\frac{e_{n}}{e_{0}}\frac{\sin kd}{\cos kd}$$

where *M*_0_ is the sensitivity of the reference hydrophone; *e*_0_ and en represent the outputs of the reference hydrophone and the tested MEMS hydrophone respectively; *d* denotes the underwater depth of the tested MEMS hydrophone and the reference hydrophone (*d*_0_ = *d*). The array MEMS hydrophone is tested in steps of 1/3 octave from 20 Hz to 1000 Hz. Figure 12 shows the curves of every structure (structure I, II, III and IV) in the array MEMS hydrophone. The curves are the sensitivities in their corresponding Xn (n = 1, 2, 3 and 4) direction of the relative coordinate system, respectively. It is with a good sensitivity of −194 ± 1 dB (@500 Hz, 0 dB ref 1 V/uPa).

### 5.2. Directivity Test

The revolver is rotated in steps of 5° from 0° to 360°. The eight outputs (every *V_x_*, *V_y_* for four units) of the array MEMS hydrophone are recorded in different angles and frequencies, and then normalized by the Equation (7).

(7) $$L=20\log D(\theta )=20\log(\frac{e_{\theta }}{e_{\max}})$$

where *e*_max_ denotes the maximum value of such output.

**Figure 13:**
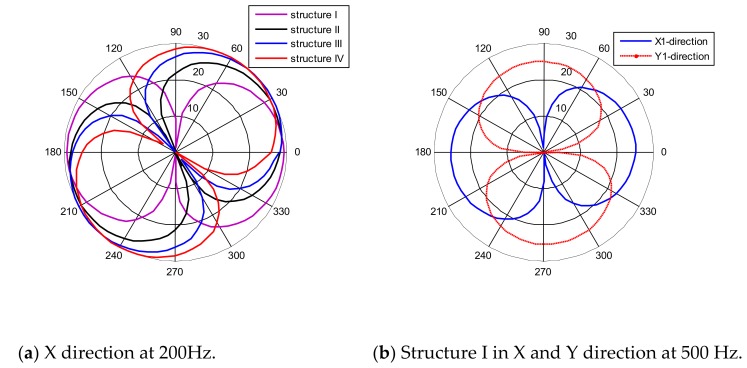
Directivity patterns of the array MEMS hydrophone.

The directivity patterns of the array MEMS hydrophone shown in Figure 13 can be obtained by the drawing of the normalized data in polar coordinates. Figure 13a shows the directivity patterns in the X-direction at 200 Hz. The directivity patterns of the array MEMS hydrophone-structure I at 500 Hz are shown in Figure 13b.

### 5.3. Positioning Test 

**Figure 14:**
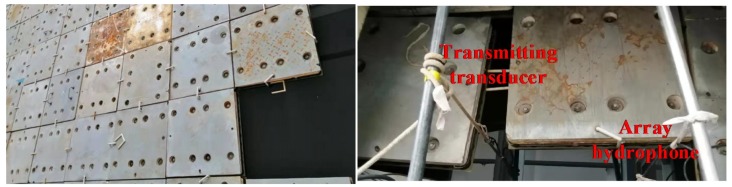
Positioning test of array MEMS hydrophone in the anechoic tank.

The positioning test of array MEMS hydrophone is carried out in the anechoic tank whose size is 20 m × 10 m × 8 m (Figure 14). A transmitting transducer is used to generate the sound signal as sound source.

**Figure 15:**
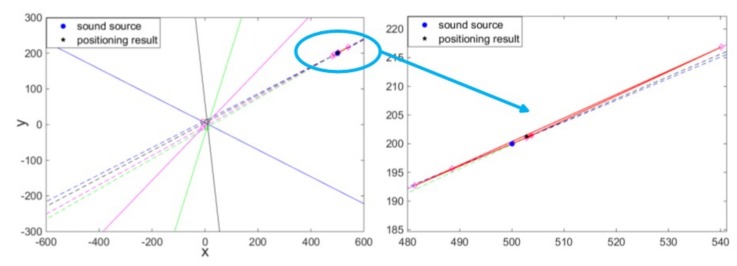
The positioning test results at (500, 200).

The center position of the array MEMS hydrophone can be seen as the origin of absolute coordinate. As an experiment example, the transmitting transducer is placed at (500, 200) whose unit is mm, in the absolute coordinate. The sound source was produced by the transmitting transducer and the hydrophone perceived it. Eight outputs of the array MEMS vector hydrophone were recorded, so the slope-intercept form y = kx + b of the eight angle lines corresponding to the four array units can be calculated, as shown in Figure 15. However, due to the error of angle information obtained by each unit of the array hydrophone, the angle lines cannot precisely intersect at a point, but an intersection area. Here, the central coordinate point (502.8, 201.3) of this area has been taken as the positioning result. It is basically consistent with the position of sound source, and the positioning error and angle error are 3.087 and 0.018, respectively.

**Figure 16:**
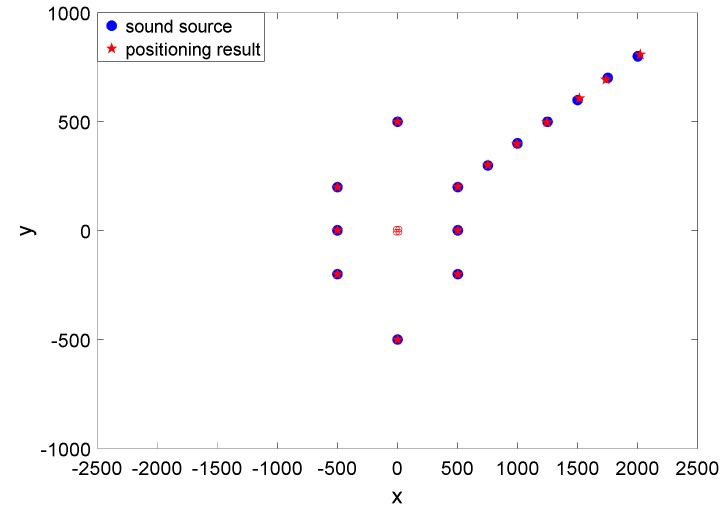
The positioning test results.

In addition, the position of the sound source has been changed, and the positioning results are shown in the Table 1 and Table 2 and Figure 16.

From the figures and tables above, it is obvious that:

(1) The frequency response performances of every unit in the array are basically the same, whose sensitivity is −194 ± 1 dB (@500 Hz, 0 dB ref 1 V/uPa). So the problem that the lower sensitivity introduced by the capacitance part (about −208 dB (@500 Hz, 0 dB ref 1 V/uPa)) [11] in the MEMS composite hydrophone can be successfully solved.
(2) In terms of directivity, every unit in the array has comparably good performance. Every pattern is smooth, and with good 8-shape directivity and orthogonality between the X and Y direction, which means that the angle measurement mismatches can be suppressed to improve the position measurement. And the maximum directivities of the units in X direction of the absolute coordinate system are respectively corresponding to the Xn (n = 1, 2, 3 and 4) direction in the relative coordinate system.
(3) Most of all, from the positioning testing results, it can be figured out that the array hydrophone can measure the position of the sound source with relatively small angle error. Naturally the left-right ambiguity problem is solved at the same time. Also, it can be found that the sound source in nπ/2 (n = 0, 1, 2 and 3) can be measured in considerably high precision. However, positioning error will be larger for further sound source. The positioning error may be reduced by combining time difference of arrival in the future.

## 6. Conclusions

This paper proposed and realized an array MEMS vector hydrophone with four different direction-angle units to solve left-right ambiguity by simultaneously measuring four sets of angle information. The working principle of the hydrophone has been analyzed. The array MEMS hydrophone has been fabricated and tested in a standing wave tube and an anechoic tank. The testing results showed that the array hydrophone exhibited a good characteristic consistency among the four units, the sensitivity of −194 ± 1 dB (@500 Hz, 0 dB ref 1 V/uPa), the working bandwidth of 20–1000 Hz, the good “8”-directivity and the good orthogonality between the X and Y direction. The array hydrophone also had a good positioning performance with the relatively small angle error, even if the sound source in the X or Y direction of the absolute coordinate system. So, not only the problem of left-right ambiguity has been solved, but also the position of the sound source can be obtained. Also, the capacitance part with the lower sensitivity in the MEMS composite hydrophone can be successfully avoided. The array MEMS hydrophone provides a new way for small-size underwater vehicles to position the sound source.

## Figures and Tables

**Table 1:** The positioning test results with different angles.

**Sound Source (mm)**	(500, 0)	(500, 200)	(0, 500)	(−500, 200)	(−500, 0)	(−500, −200)	(0, −500)	(500, −200)
**Positioning Result (mm)**	(501.2, 0.3)	(502.8, 201.3)	−1.3, 498.8)	(−499.5, 199.7)	(−499.3, 0.3)	(−499.8, −199.5)	(−1.8, −499.8)	(502.9, −200.8)
**Positioning Error (mm)**	1.237	3.087	1.769	0.583	0.762	0.539	1.811	3.008
**Angle Error (°)**	0.034	0.018	0.149	0.010	0.034	0.042	0.206	0.035

**Table 2:** The positioning test results with different-distance at θ=21.801.

**Sound Source (mm)**	(500, 200)	(750, 300)	(1000, 400)	(1250, 500)	(1500, 600)	(1750, 700)	(2000, 800)
**Positioning Result (mm)**	(502.8, 201.3)	(753.9, 301.6)	(995.3, 398.5)	(1243.3, 498.6)	(1514.1, 606.3)	(1732.7, 693.5)	(2023.1, 808.7)
**Positioning Error (mm)**	3.087	4.215	4.934	6.845	15.443	18.481	24.684
**Angle Error (°)**	0.018	0.003	0.019	0.051	0.022	0.012	0.013
